# Pediatric brain tumors: A neuropathologist's approach to the integrated diagnosis

**DOI:** 10.3389/fped.2023.1143363

**Published:** 2023-03-08

**Authors:** Angela N. Viaene

**Affiliations:** ^1^ Department of Pathology and Laboratory Medicine, Children’s Hospital of Philadelphia, Philadelphia, PA, United States; ^2^ Department of Pathology and Laboratory Medicine, University of Pennsylvania Perelman School of Medicine, Philadelphia, PA, United States

**Keywords:** integrated diagnosis, glioma, molecular, central nervous system, pediatric, brain, tumor

## Abstract

The classification of tumors of the central nervous system (CNS) is a rapidly evolving field. While tumors were historically classified on the basis of morphology, the recent integration of molecular information has greatly refined this process. In some instances, molecular alterations provide significant prognostic implications beyond what can be ascertained by morphologic examination alone. Additionally, tumors may harbor molecular alterations that provide a therapeutic target. Pediatric CNS tumors, in particular, rely heavily on the integration of molecular data with histologic, clinical, and radiographic features to reach the most accurate diagnosis. This review aims to provide insight into a neuropathologist’s approach to the clinical workup of pediatric brain tumors with an ultimate goal of reaching an integrated diagnosis that provides the most accurate classification and informs prognosis and therapy selection. The primary focus will center on how histology and molecular findings are used in combination with clinical and radiographic information to reach a final, integrated diagnosis.

## Introduction and historical perspective on tumor classification

Historically, central nervous system (CNS) tumors were classified based on their presumed cell of origin and their developmental differentiation states. Several tumor names that persist in the current World Health Organization (WHO) Classification of CNS Tumors, 5th edition (1) are based on this principle. Entities such as astrocytomas and oligodendrogliomas, once thought to be derived from and/or composed of neoplastic astrocytes and oligodendrocytes, respectively, are examples of this type of nomenclature. Recent studies have shown the picture to be much more complicated. For example, experimentally, astrocytomas can arise from oligodendrocyte precursor cells, neural precursor cells, and astrocytes (2–4). Single cell RNA sequencing studies of human gliomas found that IDH-mutant astrocytomas and oligodendrogliomas show gene expression signatures similar to neural precursor-like cells as well as both astrocytic and oligodendroglial lineages (5, 6).

Light microscopic evaluation of hematoxylin and eosin (H&E) stained sections was the historical basis of histologic classification and continues to play a fundamental role today. Additionally, various ancillary studies have been employed to aid in tumor classification. In the past, electron microscopy was utilized to assess ultrastructural features of tumors but is now rarely used in clinical practice as quicker and more economical methods are available. Immunohistochemical stains have been a routine part of tumor classification for decades and remain an incredibly useful tool today, not only for determination of cell lineages, but also as rapid, cost-effective surrogates to molecular studies (see *Molecular Testing* below).

In addition to classifying tumors, histologic evaluation was historically the sole means by which tumors were graded (a means to predict a tumor's biologic behavior). Tumors of the CNS are graded 1 through 4 with grade 1 being low-grade, and grade 4 considered to be malignant. Criteria for grading vary across tumor types though features such as elevated mitotic activity and focal necrosis are generally associated with higher-grade tumors. While histology remains the primary means for grading many CNS tumors, others are graded based on their molecular profiles. For example, diffuse midline glioma, H3 K27-altered receives a CNS WHO grade 4 regardless of histologic grade, due to the poor prognosis associated with this molecular alteration (7).

The concept of incorporating molecular information with tumor histology to form an “integrated diagnosis” was introduced in the International Society of Neuropathology (ISN)-Haarlem guidelines in 2014 (8) and formally adopted in the revised fourth edition of the WHO CNS tumor classification (9). It has since been widely expanded in the 5th edition of the WHO classification (1). This article describes a neuropathologist's approach to classifying pediatric brain tumors including how histology and molecular findings are combined with the clinical and radiographic information to reach an integrated diagnosis.

## The integrated diagnosis

The integrated diagnosis was designed to incorporate multiple critical data types (i.e., tumor type, relevant molecular information, and tumor grade) into a single, line diagnosis. To facilitate the reporting of the integrated diagnosis, a layered or tiered approach to the pathology report is often adopted and is endorsed by the ISN Haarlem consensus guidelines and the International Collaboration on Cancer Reporting (8, 10). This format frequently includes four lines: the integrated diagnosis, histologic classification, tumor grade, and molecular information. An example of a layered/tiered diagnosis follows:

Brain, right frontal lobe, tumor resection:

Integrated diagnosis: Diffuse hemispheric glioma, H3 G34-mutant, CNS WHO grade 4
Histologic Diagnosis: High-grade glioma
CNS WHO Grade: 4
Molecular Information: Alterations in *H3F3A* c.103G > A p.G35R (G34R), *ATRX* c.1144G > T p.E382*, and *TP53* c.818G > A p.R273H (Next Generation Sequencing); Positive for MGMT promoter methylation (real time PCR)

The layered diagnosis provides a means to summarize all relevant information on a given tumor. A header should be present above the four diagnostic lines which indicates the location of the tumor and the surgical procedure performed (e.g., biopsy or resection). The first line contains the integrated diagnosis which is the overall tumor classification using WHO nomenclature. Depending on the tumor type, this will often include the most relevant molecular finding(s) which are diagnostic of a given entity (e.g., the defining H3 G34 mutation in the above example). Information regarding the tumor histology and grade can be found below the integrated diagnosis. The final line is used to detail the molecular findings, including alterations which may not appear in the integrated diagnosis line but are nevertheless important information and may inform therapy (e.g., MGMT promoter methylation). Different formats for reporting the integrated diagnosis may be adopted as long as all relevant information is included. The following sections describe the process of reaching an integrated diagnosis, including histologic evaluation, molecular testing, and correlation with relevant clinical and radiographic findings.

## Histologic diagnosis and grading

Two components of the layered/tiered report format are histologic diagnosis and tumor grade. Despite the many advances in molecular diagnostics, histologic assessment remains a critical component in the workup of CNS tumors. As some molecular tests take days to weeks to result, the histologic diagnosis and grade can inform the initial treatment planning and patient/family counseling while waiting for the integrated diagnosis to be finalized. Additionally, histologic assessment is often crucial in deciding which type(s) of ancillary molecular testing is/are necessary for diagnosis, if any at all (see section on *Molecular Testing*).

Histologic diagnosis and grading are typically based on analysis of H&E stained sections often in conjunction with ancillary immunohistochemical stains. The first step in histologic evaluation is confirmation of the presence of lesional tissue, after which an abnormality is determined to be neoplastic or non-neoplastic. Tumors can be primary (originating within the CNS) or secondary (metastatic). In the pediatric population, the vast majority of tumors are primary CNS neoplasms though care must be taken to confirm this, particularly in patients with a known primary tumor outside the CNS. A primary CNS tumor is then placed into a category; examples include (but are not limited to) glial/glioneuronal, embryonal, choroid plexus tumors, germ cell tumors, nerve sheath tumors, pineal tumors, and mesenchymal neoplasms. Each of these categories contains several entities and sometimes even further differentiation into subgroups of an entity. For example, medulloblastomas are a type of embryonal tumor for which there are distinct molecular and histologic subtypes.

**Figure 1:**
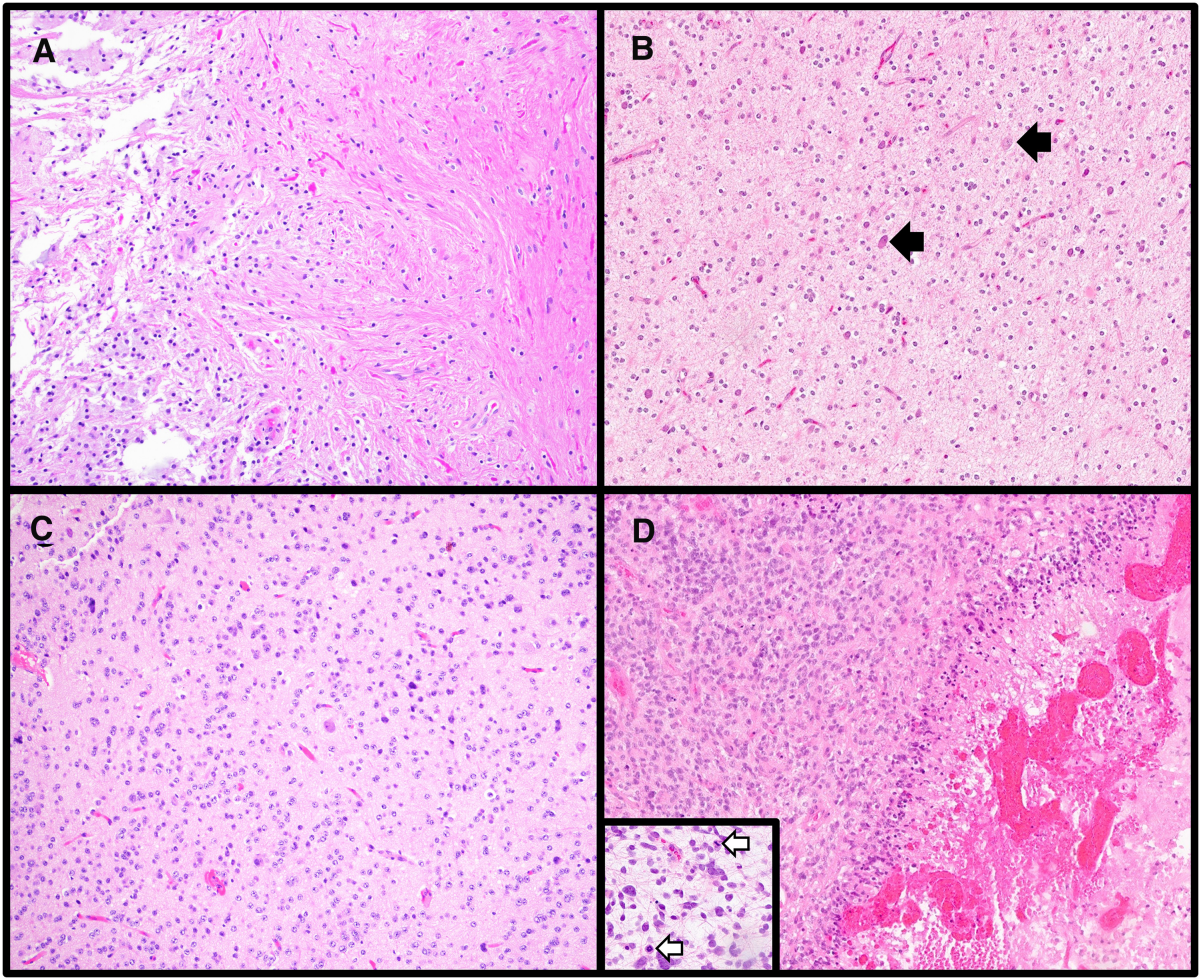
Varied histologic features of central nervous system tumors. (**A**) Pilocytic astrocytoma is a circumscribed astrocytoma with characteristic features including alternating compact (right side of image) and loose (left side of image) regions, cells with elongated (piloid processes), and Rosenthal fibers and eosinophilic granular bodies (H&E stain, 100× magnification). (**B**) Many low-grade glial/glioneuronal tumors share overlapping histologic features, including the presence of oligodendroglial-like cells as was seen in this case. A few clusters of tumor cells as well as scattered neurons (black arrows) were present. The histologic differential included multiple low-grade entities, therefore a preliminary diagnosis of low-grade glial/glioneuronal tumor pending molecular workup was rendered. The tumor was subsequently found to have an internal tandem duplication involving *FGFR1*, consistent with a diagnosis of dysembryoplastic neuroepithelial tumor (H&E stain, 100× magnification). (**C**) Diffuse glioneuronal tumor with oligodendroglioma-like features and nuclear clusters is a newly described entity for which molecular confirmation of the diagnosis *via* methylation profiling is required (H&E stain, 100× magnification). (**D**) High-grade gliomas can have varied morphologies; however, high mitotic rates (mitoses highlighted by white arrows in the inset) and focal necrosis (right side of the main image) are frequently encountered in these tumors (Diffuse midline gliomas, H3 K27-altered, H&E stains, main image 100× magnification, inset 200× magnification).

Whenever possible, the most specific histologic diagnosis is rendered. This is generally most feasible when a tumor has a distinct, “classic” morphologic appearance. For example, a well-circumscribed cerebellar tumor with alternating loose and compact regions containing scattered Rosenthal fibers composed of tumor cells with mild nuclear atypia and elongated cytoplasmic processes could confidently be given a histologic diagnosis and grade of pilocytic astrocytoma, CNS WHO grade 1 (Figure 1A). There may be instances when a specific histologic diagnosis and grade are unable to be rendered and a more generic categorization is given at the time of initial reporting in the histologic diagnosis line of the tiered report. A pathologist may feel a generic diagnosis most appropriate when a tumor does not conform to the “classic” morphologic description for a WHO entity and/or does not have adequate tissue to fully assess tumor morphology as may occur in the setting of small biopsies.

Low-grade glial/glioneuronal tumors are notorious for having overlapping histologic features, and if a tumor does not demonstrate the characteristic features of a defined entity and/or shares morphologic patterns of more than one tumor type, a more generic diagnosis of “low-grade glial/glioneuronal tumor” is sometimes adopted (Figure 1B). Ancillary molecular testing is occasionally helpful in further categorizing such tumors, though this cannot always be relied upon as glial/glioneuronal tumors also frequently harbor overlapping molecular alterations such as *BRAF* p.V600E.

Another reason for a more generic initial histologic diagnosis may be that definitive tumor classification requires molecular testing. A new WHO entity, diffuse glioneuronal tumor with oligodendroglioma-like features and nuclear clusters (DGONC), is a tumor which shares histologic overlap with oligodendrogliomas and other glial/glioneuronal tumors and cannot be diagnosed on the basis of histology and immunohistochemistry alone (Figure 1C). DGONCs lack the *IDH*-mutation and 1p/19q-codeletion seen in oligodendrogliomas and require methylation profiling for definitive classification. Even when a precise histologic diagnosis cannot be provided, it is nevertheless helpful for the pathologist to place the tumor into a broader category (e.g., glial vs. embryonal) and further refine the diagnosis as molecular information becomes available.

Grading is highly dependent on tumor type. While histologic features such as high mitotic activity and focal necrosis are features of higher-grade tumors (Figure 1D), each tumor type has its own grading criteria. For example, when using mitotic activity to grade tumors, cut-offs vary amongst different tumor entities. While ≥5 mitoses per 10 high power fields (HPF) is associated with a grade 3 (formerly termed “anaplastic”) pleomorphic xanthoastrocytoma (PXA), ≥6 mitoses per 10 HPF is suggestive of CNS WHO grade 3 oligodendroglioma, and ≥20 mitoses per 10 HPF would warrant a grade 3 for meningiomas. Additionally, not all tumor types can be assigned the full range of CNS WHO grades (1 through 4). While tumors of the pineal gland can be assigned grade 1 (pineocytoma), grades 2/3 (pineal parenchymal tumor of intermediate differentiation), and grade 4 (pineoblastoma), supratentorial and posterior fossa ependymomas are either grade 2 or 3 (once termed ependymoma and anaplastic ependymoma, respectively). To further complicate grading, there are some tumors for which molecular alterations supersede histologic grading. Within the realm of pediatric CNS tumors, one such entity is diffuse midline glioma, H3 K27-altered (DMG). DMG are infiltrating astrocytic tumors which can vary in histologic appearance from low-grade to high-grade. Regardless of histologic grade, if an infiltrative astrocytic tumor located in the midline is demonstrated to contain an H3 K27 alteration, this warrants a diagnosis of DMG (11) and an automatic CNS WHO grade of 4. For DMG and other tumors for which molecular findings determine the overall tumor grade, it has been demonstrated that the presence of a certain molecular alteration is more predictive of outcome than histologic grading (7, 12). Finally, it is important to note that there is a subset of WHO entities which are not currently assigned a grade. These may be emerging entities for which there is insufficient information regarding prognosis to accurately give a formal tumor grade.

## Molecular testing

Molecular testing is a key component to the integrated diagnosis and overall characterization of many CNS tumors. However, not all tumors require the same molecular tests, and initial histologic evaluation plays a crucial role in determining subsequent molecular workup. After formulating a differential based on histology, a pathologist will select appropriate testing for classifying the tumor, including molecular tests required for an integrated diagnosis. There are many approaches that can be taken to molecular testing which are often dependent on the suspected tumor type and/or resources availability. Stepwise approaches to molecular testing of pediatric glial, ependymal, and embryonal tumors have been previously described (13–18). Here, histology, patient age, and tumor location play a role in determining the order in which test(s) are preformed, starting with targeted techniques aimed to detect molecular alterations most commonly seen for a given tumor entity. A variety of molecular techniques can be used in this stepwise approach including immunohistochemistry, fluorescence *in situ* hybridization (FISH), and polymerase chain reaction (PCR). A benefit to this approach is that these tests are often more cost-effective and have shorter turn-around times than other molecular tests. A drawback is that some non-canonical alterations and alterations not required for diagnosis may go undetected. An alternative tactic relies on techniques which allow for the detection of a variety of alterations all in one platform (a single method approach). One such example is Next Generation Sequencing (NGS). NGS panels enable parallel sequencing of up to hundreds of genes, allowing for the detection of alterations (both mutations and fusions) most commonly found in pediatric CNS tumors. A single method approach can be advantageous for tumors which are difficult to classify histologically and/or for tumors with non-canonical alterations. Drawbacks to NGS include high tissue quality requirements, cost, and generally longer turn-around-times (up to two to four weeks). Which approach is taken may depend on the initial histologic impression, amount of tissue available for testing (small biopsy vs. large resection), and resources available to the ordering pathologist.

**Figure 2:**
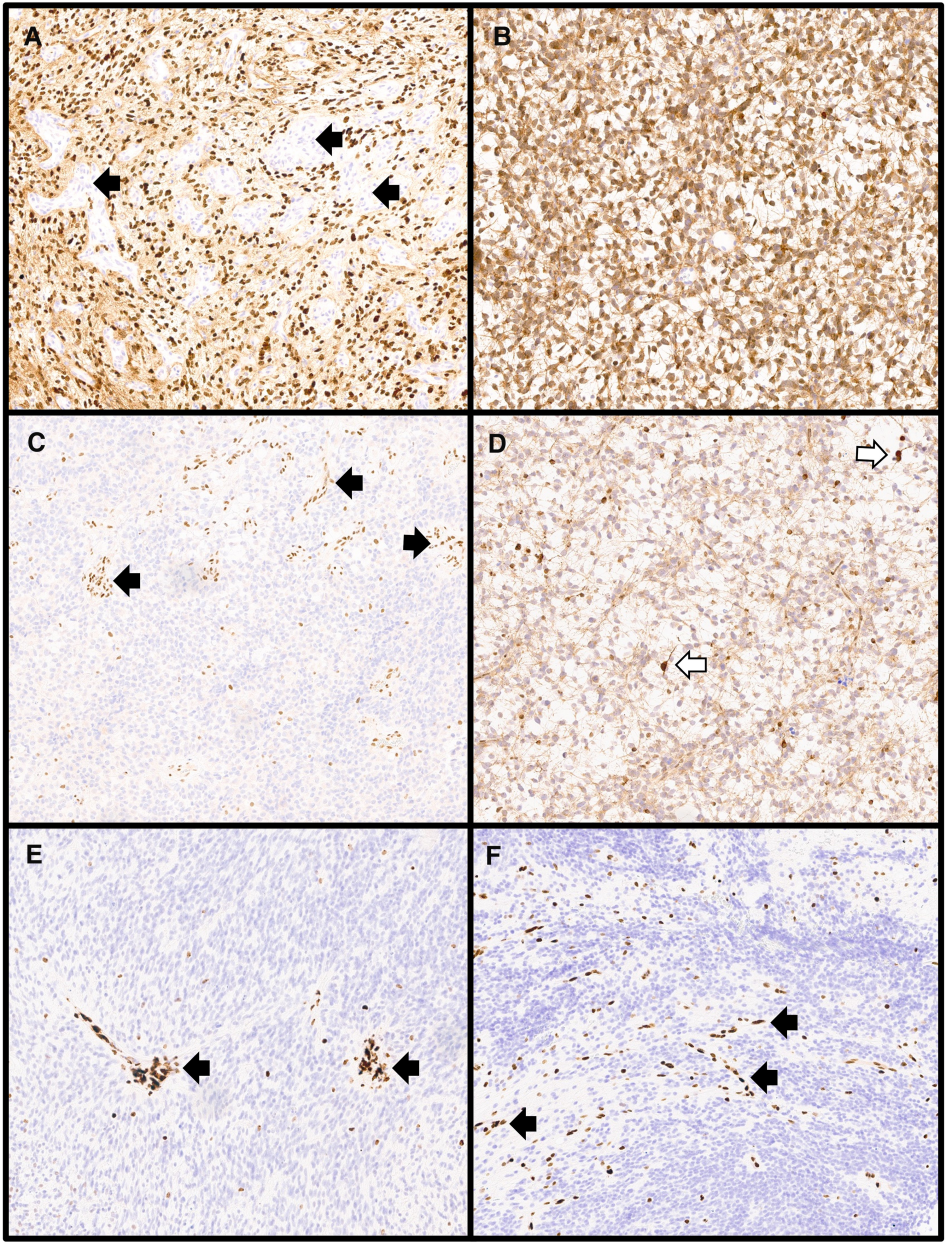
Immunohistochemical stains used in the molecular workup of central nervous system tumors. (**A**) Diffuse midline gliomas harboring the canonical H3 p.K28M (H3 p.K27M) alteration show diffuse nuclear positivity for the H3K27M immunostain while entrapped normal tissues including vessels (black arrows) are negative (H3K27M immunostain, 100× magnification). (**B**) IDH-mutant gliomas with the most common mutation (IDH1 p.R132H) will show cytoplasmic positivity for the IDH1-R132H immunostain as was seen in this astrocytoma, IDH-mutant, CNS WHO grade 3 (IDH1-R132H immunostain, 100× magnification). (**C**) Atypical teratoid/rhabdoid tumors with alterations in *SMARCB1* show loss of nuclear staining for INI1 with retained staining in non-neoplastic tissues including vessels (black arrows) (INI1 immunostain, 100× magnification). (**D**) Mutations in *ATRX* can be observed in several gliomas including astrocytoma, IDH-mutant as shown here. Alterations result in loss of nuclear ATRX immunostaining while retained nuclear staining is present in cells with the wildtype ATRX protein, including entrapped non-neoplastic glial cells (white arrows) (ATRX immunostain, 100× magnification). Loss of nuclear staining in the tumor nuclei for H3 p.K28me3 (H3K27me3) is seen in diffuse midline gliomas, H3 K27-altered, including those with non-canonical H3 p.K28M (H3 p.K27M) alterations (**E**: H3K27me3 immunostain, 100× magnification) and in posterior fossa group A ependymomas (**F**: H3K27me3 immunostain, 100× magnification) with retained staining in vessels (black arrows).

Even when a single method approach to molecular testing is used, a pathologist will often perform immunohistochemical stains as part of the initial histologic workup, including stains that detect specific molecular alterations. Immunohistochemistry is robust, affordable, and efficient (typically taking less than 24 h to complete). A variety of targeted immunohistochemical stains are available. Some of the more commonly used stains include those which detect mutant proteins for H3 p.K28M (p.K27M) (19), IDH1 p.R132H (20), BRAF p.V600E (21), and H3G34R/V (22) where positive staining indicates the presence of the mutation (Figures 2A,B). Additionally, stains for proteins including as INI-1 (23), BRG-1 (24), and ATRX (25) can be used to detect molecular alterations in the genes encoding those proteins (Figures 2C,D). For these stains, staining is retained in cells with the wild-type protein and lost in cells that harbor the mutation. Immunohistochemical staining is an excellent first-line method for determining the molecular subgroups for medulloblastomas (26). A small panel of stains for beta-catenin, YAP1, and GAB1 can be used to identify WNT-activated (positive nuclear staining for beta-catenin and positive staining for YAP1) and SHH-activated tumors (positive for YAP1 and GAB1). Group 3/4 tumors are negative for these stains. In some instances, genetic alterations affect histone methylation which can be detected *via* immunohistochemical staining. Loss of trimethylation of the lysine 27 residue on histone 3 (H3K27me3) is seen in the setting of DMG, including tumors which lack the canonical H3 p.K28M (K27M) mutation (27–30). H3K27me3 is also a useful immunohistochemical stain for distinguishing group A posterior fossa ependymomas (PFA) from group B (PFB) (Figures 2E,F). PFA demonstrate a lack of H3K27me3 staining while it is retained in PFB (31). Given the utility of immunohistochemical stains in the workup of CNS tumors, stains will likely continue to be developed in the future to aid in tumor classification.

In recent years, the utility of methylation profiling in the diagnosis of CNS tumors has been established (32, 33). This technique involves using epigenomic methylation patterns to differentiate and classify CNS neoplasms. Methylation profiling was shown to be an effective way to classify ependymomas (34); providing prognostic information beyond what could be predicted based upon histology alone. Additionally, for posterior fossa ependymomas for which there are no defining mutations or fusions, methylation profiling was able to delineate two distinct groups, PFA and PFB, which have different clinical outcomes (34, 35). Methylation profiling can be used to distinguish subgroups of embryonal tumors including medulloblastoma (36–38) and atypical teratoid/rhabdoid tumors (AT/RT) (39, 40). In the 5th edition of the WHO classification of CNS tumors, methylation profiling is listed under “desirable diagnostic criteria” for the diagnosis of several tumor types, especially in instances where a final tumor classification is unable to be reached on the basis of histologic examination and other molecular techniques (termed “unresolved cases”). Finally, for a small subset of entities such as DGONC (41) and high-grade astrocytoma with piloid features (HGAP) (42), methylation is an “essential diagnostic criterion” for tumor classification (see *Forming the Integrated Diagnosis* section below for discussion of WHO “essential” and “desirable” diagnostic criteria). A pathologist will often choose to employ methylation profiling for diagnostically challenging cases and for those in which the leading differential includes a tumor for which methylation testing is listed under the “essential diagnostic criteria”. However, the results of methylation profiling need to always be interpreted in the context of histologic and other molecular findings. A given tumor may show different matches across various classifiers or different versions of the same classifier. In some cases, a match may not be found. Nevertheless, in many instances, methylation profiling provides yet another powerful tool in the classifications of CNS tumors.

## The importance of clinical and radiographic features

Even with the many advances in imaging techniques, histologic examination is still considered to be the gold standard for CNS tumor diagnosis. However, the importance of radiographic features in tumor classification cannot be understated. Gross (macroscopic) examination is an essential part of anatomic pathology as gross features are used in conjunction with microscopic findings to construct a complete pathology report. For example, in a resection for colonic adenocarcinoma, gross examination is used to document essential features including tumor size, location, and distance from margins of resection. Gross examination is also used to determine which parts of a specimen are sampled for microscopic examination. For colonic adenocarcinoma, the tumor, margins, uninvolved bowel, and lymph nodes are sampled for microscopic examination in order to accurately classify and stage the tumor. The same techniques cannot be applied to CNS tumors; specimens are often received in multiple fragments and little-to-no normal surrounding CNS parenchyma is present. For tumors which are located in unresectable locations, small biopsies are procured which may not be representative of the tumor as a whole. Therefore, as a pathologist cannot rely as heavily on gross examination in these instances, radiographic features are of great importance.

**Figure 3:**
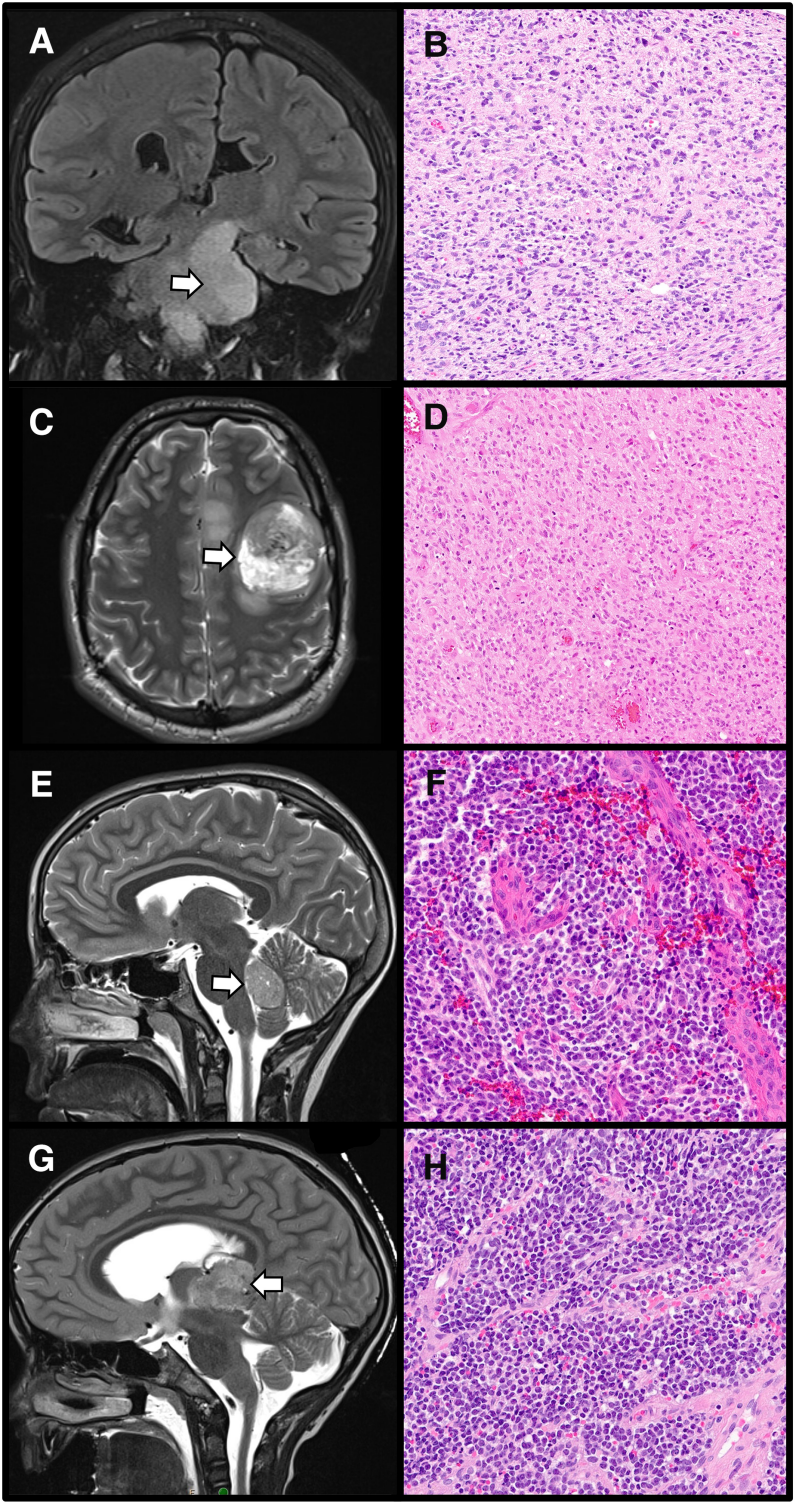
The importance of radiographic localization for central nervous system tumors. A diffuse midline glioma, H3 K27-altered, appearing as an ill-defined left brainstem T2 hyperintensity within the required midline location (**A**: Coronal T2-weighted MRI, arrow designates tumor) with corresponding histology demonstrating an infiltrative, cellular tumor with nuclear atypia (**B**: H&E stain, 100× magnification). In contrast, diffuse hemispheric gliomas, H3 G34-mutant are located within the cerebral hemispheres (**C**: Axial T2-weighted MRI, arrow designates tumor). These tumors show high-grade histology (**D**: H&E stain, 100× magnification). Medulloblastomas are embryonal tumors arising within the posterior fossa (**E**: Sagittal T2-weighted MRI, arrow designates tumor) which demonstrate poorly differentiated cells, often with a high nuclear to cytoplasmic ratio, and high mitotic activity (**F**: H&E stain, 200× magnification). Location within the pineal region (**G**: Sagittal T2-weighted MRI, arrow designates tumor) is required for a diagnosis of pineoblastoma, an embryonal tumor which histologically resembles other embryonal neoplasms arising outside the pineal parenchyma (**H**: H&E stain, 200× magnification).

The diagnosis of CNS tumors should take into account the tumor location (Figure 3). Entities such as DMG are tumors of the midline; a tumor which has no association with the midline cannot receive this diagnosis (1, 11). Tumors which do not arise in the posterior fossa should not be classified as medulloblastomas (1). Diffuse hemispheric glioma, H3 G34-mutant is a tumor for which location within the cerebral hemispheres is considered an “essential diagnostic criterion” (1). Documentation of tumor location within the pineal is essential for diagnosis of pineal parenchymal tumors including pineoblastoma which has morphologic overlap with other embryonal tumors (compare Figures 3F,H) (1). Therefore, when forming a final histologic diagnosis, care must be taken to ensure tumor location is compatible with what is described for a brain tumor entity.

Tumor location can also be extremely useful in forming a differential and determining initial molecular testing in a stepwise approach (see *Molecular Testing* above) as tumors in different CNS compartments have distinct molecular features. In the case of a pediatric diffuse high-grade glioma, a pathologist would look for H3 K28 (H3 K27) alterations in tumors of the midline and H3 G34R/V alterations in hemispheric tumors (18). Low-grade gliomas of the midline are more likely to harbor *BRAF* fusions whereas hemispheric tumors are more likely to have *BRAF* p.V600E alterations (14) which may inform initial molecular test selection. While ependymomas often have similar histologic features regardless of location, their molecular alterations are distinct with *ZFTA* and *YAP1* fusions seen in supratentorial ependymomas and *MYCN* amplification in a subset of spinal ependymomas. As described above, in some instances like DMG, molecular findings must be interpreted in the context of tumor location.

Evidence of infiltration (radiographic and/or histologic) is also an essential component in the diagnosis of some gliomas. As stated within the entity name, DMG are diffuse; gliomas which are considered to be circumscribed cannot be diagnosed as DMG, even when they harbor H3 K28 (H3 K27) alterations (11, 43). As DMG occur in locations not amenable to resection, this entity is typically biopsied. When histologic evidence of tumor infiltration is not apparent, radiographic findings can be essential in supporting the diagnosis. Similarly, for some low-grade, diffuse gliomas which can have morphologic overlap with circumscribed gliomas, radiographic evidence of diffuse growth can be very helpful in categorizing the tumor, particularly when limited tissue is available for histologic evaluation.

Correlation with post-operative imaging can also be incredibly useful to pathologists when the histologic appearance of a lesion does not correspond to what was suspected radiographically. Discordant findings, particularly in small biopsies of larger lesions, may occur due to sampling. Post-operative imaging evaluation of the biopsy site within the lesion of interest is critical. If the radiographic features appear high-grade while the histologic findings appear low-grade or even non-lesional, biopsy location at the edge of or adjacent to a tumor may explain this discordance. If there is no histologic evidence of a lesion that was apparent radiographically, pathologists should correlate with post-operative imaging to confirm that gross total resection or evidence of biopsy within the lesion was achieved. Imaging confirmation that lesional tissue was procured should prompt further histologic examination *via* deeper sectioning and often molecular workup.

Patient age is another important piece of information pathologists use when forming an initial differential. Some tumors have a strong association with presentation in infancy including desmoplastic infantile ganglioglioma/desmoplastic infantile astrocytomas (44) and infant-type hemispheric gliomas (45). Diffuse hemispheric glioma H3 G34-mutant, on the other hand, is more likely to be diagnosed in adolescent/young adult patients (46). In high-grade gliomas in particular, patient age plays an important role in the prioritization of molecular testing when using a stepwise approach (18).

One final piece of clinical information to consider is whether the patient has a genetic tumor predisposition syndrome. Syndromes which may be encountered and are associated with CNS tumors include Li-Fraumeni syndrome, constitutional (biallelic) mismatch repair deficiency syndrome (CMMRD), Lynch syndrome, *DICER1* syndrome, rhabdoid tumor predisposition syndrome, familial adenomatous polyposis, Gorlin syndrome, tuberous sclerosis, neurofibromatosis type 1 and type 2, and von Hippel-Lindau syndrome. In some instances, this may be known or suspected prior to surgery. With increasing use of paired tumor-normal NGS, germline variants are being more frequently detected. Occasionally the pathologist may be the first to suspect an underlying tumor predisposition syndrome and can suggest further clinical workup to look for germline variants. It is therefore important to be aware of the associations between certain tumor types and the above syndromes. While some associations are well known, for example, plexiform neurofibromas in the setting of neurofibromatosis type 1, others may not be as apparent. In the pediatric population, high-grade gliomas with marked nuclear pleomorphism and/or multinucleated giant cells can be encountered in the setting of variants in mismatch repair genes (CMMRD and Lynch syndrome) and Li-Fraumeni syndrome (due to germline mutations in *TP53*) (18). The pathologist can perform additional immunostains for mismatch repair proteins in suspected cases (47).

## Forming the integrated diagnosis

The formulation of an integrated diagnosis should take into account all pertinent clinical, radiographic, histologic, immunohistochemical, and molecular information. As such, a final integrated diagnosis may not be able to be reached in the immediate days following surgery should more extensive molecular testing be required. In such instances, the pathologist will often issue an initial (or preliminary) report which details the histologic diagnosis and grade (two of the four lines within the layered/tiered format). This is useful to inform initial clinical decision making though care should be taken to note that molecular findings may alter the final tumor diagnosis and/or grade. Once the full workup is complete, the integrated diagnosis can be finalized and reported.

In many cases, the clinical, histologic, and molecular data are all compatible, meeting the “essential diagnostic criteria” for a defined WHO entity, and a final integrated diagnosis can be reached without difficulty. “Essential diagnostic criteria” are those which are considered “must-have features” in order to diagnose a specific entity according to the 5th edition of the WHO classification of CNS tumors (1). The WHO classification also lists “desirable diagnostic criteria” for each CNS tumor which are those that “support a diagnosis but are not mandatory”. For example, the “essential diagnostic criteria” for diffuse low-grade glioma, MAPK pathway-altered are as follows: diffuse glioma with absent or minimal mitotic activity, neither microvascular proliferation nor necrosis, a genetic alteration in the MAPK pathway, IDH-wildtype and H3-wiltype status, and absence of homozygous deletion of *CDK2NA*. “Desirable diagnostic criteria” for this entity are: onset in childhood, adolescence, or early adulthood and the absence of morphologic features or DNA methylation profile suggestive of an alternative tumor type in which *FGFR* or *BRAF* abnormalities occur (1). As evidenced by these criteria, the diagnosis for diffuse low-grade glioma, MAPK pathway-altered relies on the integration of histologic and molecular findings with clinical features (age of presentation) supporting the diagnosis. Radiographic findings such as tumor location can also be listed as “essential diagnostic criteria”. Examples include pediatric-type diffuse high-grade gliomas (i.e., hemispheric location for diffuse hemispheric glioma, H3 G34-mutant and midline location for diffuse midline glioma, H3 K27-altered), neurocytomas (i.e., intraventricular location for intraventricular neurocytoma and extraventricular location for extraventricular neurocytoma), ependymomas (supratentorial, posterior fossa, and spinal), and pineal parenchymal tumors (pineal region location).

Tumors meeting the required “essential diagnostic criteria” can be given a specific WHO diagnosis. There are instances, however, in which this is not possible. The terms “not elsewhere specified (NOS)” and “not elsewhere classified (NEC)” were introduced in cIMPACT-NOW update 1 (48) and endorsed in the 5th edition of the WHO classification of CNS tumors (1) for these occurrences. The NOS distinction applies when diagnostic information required for a specific entity is not available. This may occur when the required molecular testing was not performed or when molecular testing failed (e.g., an insufficient specimen). The NEC designation is used for tumors when necessary diagnostic testing was performed and was successful, but the results do not align with a specific WHO entity. This can occur when the clinical, histologic, immunohistochemical, and/or molecular findings are not concordant. NEC should only be used when a tumor has received an adequate pathological workup. The NEC and NOS designators are important in distinguishing tumors which do not meet WHO diagnostic criteria. For these entities, pathologists will often use a “descriptive diagnosis” (e.g., high-grade glioma, NOS or NEC) that is not an official WHO entity. These diagnoses can still be reported in the layered/tiered format, typically accompanied by a note indicating the reason for the NOS/NEC distinction.

## Conclusion

The integrated diagnosis has become central to the WHO classification of CNS tumors. It is expected that molecular findings, used in conjunction with histology and clinical information, will be further expanded in future classifications. We can no longer simply look at the tissue under the microscope and hope to render the most accurate diagnosis. Tumor classification requires integration of clinical history, radiologic findings, histology, immunohistochemistry, and often molecular findings. The ultimate goal of this integrated approach to the diagnosis of CNS tumors is to provide the most accurate representation of tumor biology and in turn, inform therapy selection and prognosis.
